# Estimating the Resources Needed and Savings Anticipated from Roll-Out of Adult Male Circumcision in Sub-Saharan Africa

**DOI:** 10.1371/journal.pone.0002679

**Published:** 2008-08-06

**Authors:** Bertran Auvert, Elliot Marseille, Eline L. Korenromp, James Lloyd-Smith, Remi Sitta, Dirk Taljaard, Carel Pretorius, Brian Williams, James G. Kahn

**Affiliations:** 1 Hopital Paul Brousse, INSERM U687, Villejuif, France; 2 Faculté de Médecine, University of Versailles, Saint Maurice, France; 3 Hôpital Ambroise Paré, APHP, Boulogne, France; 4 Philip R. Lee Institute for Health Policy Studies, University of California San Francisco, San Francisco, California, United States of America; 5 Department of Public Health, University Medical Centre Rotterdam, Rotterdam, The Netherlands; 6 The Global Fund to Fight AIDS, Tuberculosis and Malaria, Geneva, Switzerland; 7 Department of Biology, Pennsylvania State University, University Park, Pennsylvania, United States of America; 8 Progressus, Johannesburg, South Africa; 9 SACEMA, Stellenbosch, South Africa; 10 STB/PME, WHO, Geneva, Switzerland; University of Cape Town, South Africa

## Abstract

**Background:**

Trials in Africa indicate that medical adult male circumcision (MAMC) reduces the risk of HIV by 60%. MAMC may avert 2 to 8 million HIV infections over 20 years in sub-Saharan Africa and cost less than treating those who would have been infected. This paper estimates the financial and human resources required to roll out MAMC and the net savings due to reduced infections.

**Methods:**

We developed a model which included costing, demography and HIV epidemiology. We used it to investigate 14 countries in sub-Saharan Africa where the prevalence of male circumcision was lower than 80% and HIV prevalence among adults was higher than 5%, in addition to Uganda and the Nyanza province in Kenya. We assumed that the roll-out would take 5 years and lead to an MC prevalence among adult males of 85%. We also assumed that surgery would be done as it was in the trials. We calculated public program cost, number of full-time circumcisers and net costs or savings when adjusting for averted HIV treatments. Costs were in USD, discounted to 2007. 95% percentile intervals (95% PI) were estimated by Monte Carlo simulations.

**Results:**

In the first 5 years the number of circumcisers needed was 2 282 (95% PI: 2 018 to 2 959), or 0.24 (95% PI: 0.21 to 0.31) per 10 000 adults. In years 6–10, the number of circumcisers needed fell to 513 (95% PI: 452 to 664). The estimated 5-year cost of rolling out MAMC in the public sector was $919 million (95% PI: 726 to 1 245). The cumulative net cost over the first 10 years was $672 million (95% PI: 437 to 1 021) and over 20 years there were net savings of $2.3 billion (95% PI: 1.4 to 3.4).

**Conclusion:**

A rapid roll-out of MAMC in sub-Saharan Africa requires substantial funding and a high number of circumcisers for the first five years. These investments are justified by MAMC's substantial health benefits and the savings accrued by averting future HIV infections. Lower ongoing costs and continued care savings suggest long-term sustainability.

## Introduction

Observational studies have repeatedly shown that male circumcision (MC) offers substantial protection against HIV infection [1]. Three randomised controlled trials (RCTs) recently confirmed these findings, with consistent estimates of effectiveness. In 2005, the first RCT of medical adult MC (MAMC), conducted in Orange Farm, South Africa, found a risk reduction between study arms of 60% (95% CI: 32 to 76) [2]. Combined with the two other RCTs conducted in Kenya and Uganda, the overall risk reduction was 58% (95% CI: 43 to 69%) [3]–[5].

The encouraging results of the Orange Farm trial prompted wide interest in Africa towards MC as an HIV prevention strategy. Currently about a third of the African male population is circumcised but the practice is less common in southern and East Africa, where the HIV epidemic is especially severe. The circumcision of adult males was shown to be more effective and cost-effective than other general population HIV prevention strategies [6]. Making MAMC a public health priority has been endorsed by international agencies such as WHO and UNAIDS [7]. In 2006, supported by UNAIDS and WHO, most sub-Saharan African countries with low MC prevalence and high HIV prevalence started preparing for the implementation of MAMC. Zambia decided not to wait for the results of the Ugandan and Kenyan trials, integrating MAMC in its national plan against HIV in the beginning of 2006.

The acceptability of MAMC appears to be high. Before the clinical trials were completed, studies were conducted in nine African countries with a high HIV prevalence and a low MC prevalence. Most uncircumcised males were willing to become circumcised (median 65%; range 29 to 87%) and most women were willing to have their sons circumcised (median 81%; range 70 to 90%) [8].

The results of the Orange Farm trial suggest that MAMC could have a substantial impact on health, with favourable economic implications. An epidemic model estimated that full coverage of MAMC in African countries where the majority of males are uncircumcised would avert 2 to 8 million HIV infections in the next 10 years [9] while a cost-effectiveness model applied to Gauteng Province, South Africa, suggested that performing 1 000 MAMCs in South Africa would avert 189 to 428 HIV infections and would save $1.3 to $3.6 million over 20 years [6].

Given the favourable evidence for the impact and feasibility of MAMC and the WHO-UNAIDS recommendations to expand access to safe male circumcision services [7], funding will likely become available for the widespread implementation of MAMC in the near future. It is thus crucial to evaluate the economic requirements and consequences for rolling out MAMC in Africa. The objectives of the present study were to estimate the human and financial resources needed for a rapid roll-out of MAMC and the net costs or savings when taking into account averted costs of HIV medical care. We focused on 14 countries with existing male circumcision prevalence lower than 80% and HIV prevalence among adults higher than 5%, since these settings have been shown to be best for MAMC to produce a large reduction in HIV with favourable economic outcomes. We also included two sites where recent MAMC trials were conducted: the Nyanza province of Kenya (the area of Kenya meeting the inclusion criteria) and Uganda.

## Methods

We developed a cost model of MAMC integrated with a demographic and HIV epidemic model. The technical details of the model are provided in Supporting Information S1, which is available as a spreadsheet upon request from the corresponding author. We ran the model for each of the 16 locations (15 countries and one province) and summed them up to obtain aggregate results. We included Botswana, Burundi, Central African Republic, Kenya's Nyanza province, Lesotho, Liberia, Malawi, Mozambique, Namibia, Rwanda, South Africa, Swaziland, Tanzania, Uganda, Zambia and Zimbabwe. In 2007, these countries had a combined total adult population (aged 15 to 49) of 96 million, or 29% (96/331) of the corresponding age group for all 42 sub-Saharan countries. The number of HIV-positive adults was estimated to be 14.6 million (59.8% of the infected people in sub-Saharan Africa, 14.6/24.3) [9]. The number (%) of uncircumcised adult males was estimated to be 30.5 million (56.4%, 30.5/54.1). South Africa provided the largest proportion of all uncircumcised adults in the sample (26.1%) (Table 1).

**Table 1:** Setting-specific model inputs (for calendar year: 2007) and cost predictions on rolling out medicalised adult male circumcision using the public cost model.

Setting	*Model input data*				*Model predictions*	
	Population aged 15 to 49 years (millions)	Prevalence of MC before MAMC intervention (percent)	Uncircumcised adults in millions (% of total)	Adult prevalence of HIV (percent)	Number of circumcisers in the first 5 years*	Funding required for MAMC programming (million US$), total for first 10 years
Botswana	0.91	25	0.34 (1.1%)	37.3	25 (18 to 37)	25 (17 to 36)
Burundi	3.41	2	1.67 (5.5%)	6.0	146 (107 to 209)	47 (32 to 64)
CAR**	1.81	67	0.3 (1%)	13.5	16 (9 to 25)	5 (3 to 8)
Kenya****	2.15	10	0.97 (3.2%)	24.0	82 (59 to 117)	30 (21 to 41)
Lesotho	0.86	0	0.43 (1.4%)	28.9	35 (26 to 51)	13 (9 to 17)
Liberia	1.62	70	0.24 (0.8%)	5.9	12 (7 to 19)	4 (2 to 6)
Malawi	5.45	17	2.26 (7.4%)	14.2	190 (142 to 285)	63 (42 to 84)
Mozambique	9.02	56	1.98 (6.5%)	12.2	130 (90 to 196)	45 (29 to 61)
Namibia	0.94	15	0.4 (1.3%)	21.3	31 (22 to 45)	19 (14 to 27)
Rwanda	3.98	10	1.79 (5.9%)	5.1	152 (114 to 225)	51 (36 to 68)
South Africa	24.47	35	7.95 (26.1%)	24.6	549 (401 to 796)	451 (313 to 649)
Swaziland	0.51	50	0.13 (0.4%)	38.8	8 (6 to 12)	5 (3 to 7)
Tanzania	18.09	70	2.71 (8.9%)	8.8	133 (74 to 220)	45 (24 to 70)
Uganda	11.64	25	4.37 (14.3%)	4.1	364 (266 to 528)	127 (87 to 169)
Zambia	4.89	12	2.15 (7.1%)	16.5	182 (134 to 264)	69 (49 to 93)
Zimbabwe	6.26	10	2.82 (9.2%)	24.6	227 (168 to 326)	78 (56 to 105)
Total	96.01	-	30.52 (100%)	-	2 282 (2 018 to 2 959)	1 077 (855 to 1 448)
Average	-	36	-	15.6	-	-

* Assuming scale-up to maximum acceptable coverage over an initial period of 5 years (see text).

** Central African Republic.

**** Province of Nyanza.

For each location we specified a population composed of individuals matched by gender, age group (child/adult), male circumcision status and HIV status. HIV-positive individuals could receive antiretroviral therapy (ART) and died of AIDS or of other causes (Figure S1). HIV transmission was simulated in a simple, dynamic, compartmental model, without consideration of strata of sexual risk behaviour [9]. To this model we added the MAMC intervention, cost parameters and details on ART.

We assumed that a given number of adults were circumcised each year, independently of their HIV status, until full coverage was achieved. We estimated the cost and effect of this intervention on HIV as a function of time. We assumed that a fixed proportion of males were already circumcised before becoming adults and that this was constant over time.

The demographic parameters and the HIV epidemic model were kept simple in order to allow for the calculation of anticipated resource needs and program cost with a reduced set of input parameters. Comparison with earlier models assured a realistic representation of the key factors related to demography, HIV and male circumcision status as well as the cost of the intervention.

The full list of input parameters is reported in Table 2 with numerical values for South Africa. Key country-specific parameters except cost are given in Table 1.

**Table 2:** Key input parameters and numerical values corresponding to the South African scenario, for the year 2007.

Demography	Value	Relative uncertainty range* (%)
Initial population size of adults in the geographical setting	24 470 000 [9]	0
Crude birth rate	1.8% per year [28]	20
Newborns reaching adulthood (age 18)	88% [29]	20
Life expectancy when becoming adult (without HIV)	47 years [30], [31]	20
Boys circumcised before reaching adulthood	35% [32]	10
**HIV epidemic model**		
Initial HIV prevalence among adults at start of intervention	24.6% [33]	20
Ratio of male-to-female and female-to-uncircumcised-male HIV transmission rates, in the absence of MC	1.5 [34], [35]	50
Reduction in female-to-male transmission of HIV due to MC	60% [2]	20
**HIV medical treatment**		
Percent of HIV-positive people receiving treatment before becoming eligible for ART	30% (AE)	20
Cost of this treatment (total per patient)	$729 US$ (AE)	50
Percent of HIV-positive people eligible for ART who receive ART	30% [36]	50
Life expectancy on ART	10 years [37]	50
Cost of ART for eligible patients (per patient per year)	993 US$ [37]	50
Percent of HIV-positive people eligible for ART who receive non anti retroviral treatment	30% (AE)	50
Cost of this treatment (lifetime total per patient)	2 743 US$ [37]	50
**MAMC program parameters**		
Duration to reach maximum male circumcision prevalence	5 years (AE)	0
Number of circumcisions per day per circumciser	10 (AE)	50
Number of working days per year	230 (AE)	20
Adult males who will remain uncircumcised	15% (AE)	50
**Public MAMC program cost**		
Initial investment per circumcision facility	28 778 US$ (AE)	50
Number of circumcisers per circumcision facility	2 (AE)	50
Initial training per circumciser	8 985 US$ (AE)	50
Salary of each circumciser	2 246 US$ per month [38]	50
Salary of health care workers/counsellors per circumciser	59% of the circumciser [38]	50
Supplies cost per patient circumcised	11 US$ [12]	50
Facility and program overheads (detail in text)	110% of all costs (AE)	50
**Private MAMC program cost**		
Circumcision cost	72 US$ (AE)	50
Annual program overhead cost	10% (AE)	50
**Miscellaneous parameters**		
Discount rate	3% per year [16]	0

The same assumptions were used for other countries and settings, except for the country-specific parameters given in Table 1 and for unit costs adjusted to reflect differences in gross domestic product per capita (see text).

AE = author's estimates.

* A range of x% for a parameter of a value v indicates that the range in which this parameter was varied from v(1−x) to v(1+x).

### Cost models

We assumed that MAMC services were intensive for the first five years, in order to achieve maximum attainable circumcision levels, and then dropped to the rate necessary to maintain these levels. It was assumed that in this “initial period” of five years a large constant annual number of MAMCs were performed, sampled randomly among all uncircumcised adult men willing to be circumcised. The prevalence of MC rose rapidly. When MC prevalence reached the specified threshold, MAMC acceptance was saturated and dropped to a lower rate. The proportion of males becoming adults who refused MAMC determined long-term MC saturation. Thus, after the initial period, the number of circumcisions performed annually was reduced to the number of male children/adolescents entering adulthood, not already adequately traditionally circumcised and willing to be circumcised. For this analysis, we assumed that current neonatal and childhood MC practices did not change. We assumed that the roll-out led to an increase in the prevalence of MC among adult males to 85% in all settings. In the sensitivity analysis, we assumed an MC prevalence increase to 55%, except for the 4 countries with an MC prevalence greater than this limit: Central African Republic, Liberia, Mozambique and Tanzania.

Program costs were composed of *initial* and *annual* costs. We explored a *public* cost scenario assuming the use of government health infrastructures only and a *private* cost scenario assuming reliance on private health care providers only.

In the ***public cost scenario***, initial costs were per circumcision facility (for medical equipment and certification) and for training circumcisers. Annual costs included the oversight and promotion of MAMC, the salaries of full-time circumcisers, surgical staff and counsellors, the direct non-salary cost of each MAMC (i.e., surgical supplies), facility overhead (i.e., operating costs) and program overhead.

The number of personnel required was based on the experience of the authors and expert opinion. In particular, we assumed that each circumciser could complete 10 circumcisions per day, as observed in recent clinical trials. For each circumciser, there was 1.0 medical assistant and 0.5 counsellor. These numbers were based on MC delivery experience in the trial in Orange Farm and were consistent with a recent study of 4 clinics in Swaziland [10]. The total salary for this 1.5 full-time equivalent was set at 59% of that of the circumciser, reflecting a salary of approximately 40% of the circumciser's, based on the WHO CHOICE health cost database for the AFRO-E region [11]. This staffing level accounted for the surgery itself plus time for follow-up, treating adverse events and counselling.

Unit cost estimates (Table 2) were derived from market data, the WHO CHOICE health cost database for the AFRO-E region [11], scientific literature, expert opinion and extrapolation from similarly structured public health programs. The costs of expendable supplies, such as drugs, anaesthesia and some instruments were based on the purchase price of 100 manufactured disposable MC kits ($11) [12]. The facility overhead costs (i.e., administration, facility maintenance, utilities) were set at 67% of the direct salary and supply costs, based on a study of 11 circumcision units in 3 countries [13]. The costs of oversight and promotion (i.e., management, communication and monitoring) were set at 26% of facility-level costs, based on a review of 9 approved “Round 6” proposals to the Global Fund to Fight AIDS, Tuberculosis and Malaria for HIV prevention programs in sub-Saharan African countries [14]. We arrived at a total overhead of 110% as the combined effect of facility and program overheads, i.e., (1.67×1.26)−1. To account for differences in price levels and salaries among countries, we adjusted most costs per health facility according to gross domestic product per capita. This resulted in sharp differences in salaries between the highest income countries (i.e., South Africa and Botswana) and other countries in this analysis. In a complementary analysis, we used the WHO CHOICE AFRO-E regional physician salary ($1 236 per month) for the lower income countries, without gross domestic product per capita adjustment. For internationally priced items (e.g., drugs), standard unit costs were used. We did not include the cost of HIV voluntary counselling and testing, since it is currently funded through other mechanisms and may not be required by many MC programs.

In the ***private cost scenario***, all facility-level costs were included in the price per MAMC paid to providers. These providers were already equipped to perform MAMC and there were no initial costs. The price of each MAMC covered salaries of circumcisers, other health staff, counselling, surgical supplies, follow-up, treatment of adverse events and operating costs. This per-circumcision price could presumably correspond to a higher unit cost than in the public scenario, due to differences in private sector costs (e.g., higher wages) or pricing strategies by providers. We spoke with informants in various settings, who provided us with a wide range of currently asked prices, from $25 in Kenya to $376 (Zambian 15 bed private clinic). In South Africa we obtained an average price of $72. In a first analysis we assumed that the cost of MAMC was $72 across all settings. This value was varied from −50% to +50% (36 to 99) in the sensitivity analysis. In addition to direct provider payments we assumed annual program overhead costs of 10% to cover the public promotion of MAMC.

To ***calculate costs and savings from HIV treatment***, we assumed that 30% of HIV-infected individuals eligible for antiretroviral treatment were receiving it. The averted cost of medical treatment for HIV over time was a function of the number of HIV infections averted each year and the rate of disease progression, combined with associated medical costs. We assigned medical costs by stages of disease, based on a study in South Africa [15], with adjustment by country for local inputs (e.g., salaries) according to *per capita* GNP, since WHO CHOICE did not provide country-level details.

The analysis adopted the perspective of a government health care payer. In the private cost scenario, the cost of each MAMC was reimbursed by the government. Since all and only direct medical costs were included, governmental and societal perspectives were similar. Program costs were discounted to 2007 at 3% per year [16]. Costs were expressed in U.S. dollars.

### Anticipated resource needs and program costs

We calculated the ***number of full-time circumcisers*** needed based on the productivity of circumcisers (circumcisions per day) and the duration of the initial period. We calculated two economic outputs. ***Program costs*** needed to roll out MAMC were calculated as the sum of the resources required to deliver a 5-year scale-up of MAMC to saturation and the resources required for the maintenance of MAMC over an additional 15 years, across all 16 settings. This 5-year period was changed to 7 and 9 years in the sensitivity analysis. ***Net costs or savings*** represented the costs of MAMC minus the savings due to averted medical care costs for HIV.

### Uncertainty range

The uncertainty of anticipated resource needs and program costs to input uncertainty was determined using Monte Carlo simulations. For each input parameter, we matched a truncated Gaussian distribution (±2 standard deviations) to the specified uncertainty range indicated in Table 2. The output range was defined as the 2.5th to 97.5th percentile interval (95% PI) among 1 000 repetitions, when all inputs were varied simultaneously.

## Results

### Number of circumcisers and HIV infections averted

Table 1 shows the number of circumcisers needed for MAMC roll-out for each individual setting. In the aggregate analysis across all 16 settings, the number of full-time circumcisers needed for MAMC roll-out over the initial, intensive 5-year phase was 2 282 (95% PI: 2 018 to 2 959). This represented 0.24 per 10 000 adults (95% PI: 0.21 to 0.31) (Table 3). Upon reaching the ‘saturation’ coverage level, in years 6 to 10, the required number of circumcisers dropped to 513 (95% PI: 452 to 664) (Table 3).

**Table 3:** Number of circumcisers required to roll out MC in 16 settings of sub-Saharan Africa, as a function of time.

	Number of circumcisers required
	Total number (95% PI) / Per 10 000 adult population (95% PI)
Years 1 to 5	2 282 (2 018–2 959) / 0.24 (0.21 to 0.31)
Years 6 to 10	513 (452 to 664) / 0.053 (0.047 to 0.069)
Years 11 to 20	567 (496 to 730) / 0.059 (0.052 to 0.076)

PI = percentile interval.

**Figure 1:**
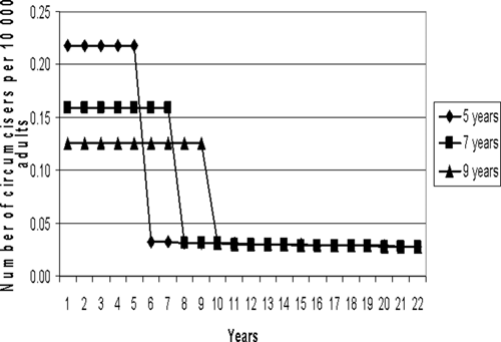
Number of circumcisers per 10 000 adults required, by year, to reach maximum MC level in 5, 7 and 9 years, respectively, in South Africa.

Figure 1 presents, for South Africa, the number of full-time circumcisers needed as a function of time when the initial period was spread over 5, 7 and 9 years. With a shorter initial period over which maximum coverage was the goal, the required number of circumcisers was highest.

Over the first 10 and 20 years of MAMC roll-out, the number of circumcisions per HIV infection averted was 10.1 (95% PI: 9.0 to 11.2) and 5.6 (95% PI: 5.1 to 6.2), respectively.

### Costs using the public scenario

Table 1 shows the program funding needed for MAMC roll-out for each individual setting using the public scenario. The bulk of costs and circumcisions was in South Africa (which has the largest national population), followed by Uganda, Zimbabwe, Zambia and Malawi (with large populations and low MC prevalence).

The program cost over the first 5 years was $919 million (95% PI: 726 to 1 245) in the public scenario (Table 4). The annual cost fell by 83% after year 5, due to lower volume of services. The program cost over 20 years was $1 347 million (95% PI: 1 070 to 1 784).

**Table 4:** Funding and net cost of rolling out MC using the public cost model, pooled across 16 countries/settings of sub-Saharan Africa.

	Public cost model
Funding (million US$) through year 5	
Total (95% PI)	919 (726 to 1 245)
Average per year (95% PI)	184 (145 to 249)
Funding (million US$) years 6–10	
Total (95% PI)	158 (126 to 204)
Average per year (95% PI)	32 (25 to 41)
Funding (million US$) through year 10	
Total (95% PI)	1 077 (855 to 1 448)
Average per year (95% PI)	108 (86 to 145)
Funding (million US$) through year 20	
Value (95% PI)	1 347 (1 070 to 1 784)
Average per year (95% PI)	67 (53 to 89)
Cost per HIV infection averted over 10 years	338 (266 to 456)
Cost per HIV infection averted over 20 years	168 (133 to 223)
Cumulative net cost* (95% PI) in million US$ at 10 years	672 (437 to 1 021)
Cumulative net cost* (95% PI) in million US$ at 20 years	−2 274 (−3 318 to −1 416)

PI = percentile interval.

* Net cost is the program cost minus savings due to averted HIV medical care (million US$; 95% percentile interval in brackets). Negative value indicates net savings.

The required MAMC program funding over the first 10 years for the 16 settings varied only slightly, between $ 919, 1 019 and 971 for the initial periods of 5, 7 and 9 years, respectively. The lower cost for the longer period of 9 years was due in part to a lower initial investment in circumcision centres and MC training.

Net costs and savings are reported in Table 4. The public scenario had a net cost over the first 10 years of $672 million (95% PI: 437 to 1 021). For South Africa this net cost was $265 million (95% PI: 121 to 471). Over a period of 20 years, there were substantial net savings of $2.3 billion (95% PI: 1.4 to 3.3). For South Africa the net savings were $960 million (95% PI: 439 to 1 522). With an MC prevalence increase among adult males to 55% instead of 85%, the cost over a 10-year period was $483 million (95% PI: 324 to 797) and the savings over a 20-year period were $1.0 billion (95% PI: 0.6 to 1.7).

Over the first 10 and 20 years of MAMC roll-out, the cost per HIV infection averted varied from $338 to $168 (Table 4). For South Africa this latter cost was $255 (95% PI: 172 to 379) over the first 20 years in the public sector.

When using the AFRO-E regional average salaries for the 14 lowest income settings (leaving South Africa and Botswana at higher levels), the program cost over 5 years was $1 279 million (95% PI: 1 074 to 1 657), slightly higher than the upper 95% PI of the main analysis. The program cost over 20 years was $1 912 million (95% PI: 1 612 to 2 438), again slightly above the main analysis upper 95% PI. The net savings over 20 years, taking into account averted HIV care, were $2 324 million (95% PI: 1 356 to 3 350). This value is similar to what was obtained in the main analysis despite a higher program cost, due to higher averted HIV care costs using the AFRO-E regional salaries.

### Costs using the private scenario

With a private cost set at $72 ($79.2 with overhead), the program cost over 5 years was $1 961 million (95% PI: 1 612 to 2 298) and $3 011 million (95% PI: 2 499 to 3 527) over 20 years. Net savings at 20 years were $610 million (265 to 1 676). With a cost per MC of $32 (excluding 10% program overhead), the program cost over 20 years in the private and public sector was the same. A net cost over 20 years of $0 was achieved at a private cost of $87 per AMC (excluding 10% program overhead).

## Discussion

This study provides the first estimates of the cost of scaling up MAMC in sub-Saharan Africa, the number of circumcisers needed and the likely savings due to averted HIV medical care costs. After numerous observational studies, three randomised controlled studies, a modelling study and a cost-effectiveness study, this analysis provides further evidence supporting the rapid roll-out of MC in sub-Saharan Africa. Cumulative net costs at 20 years are negative for the base case and for almost all sensitivity analyses, indicating that the intervention will save money.

This study has some limitations. On the costing/demand aspect, the predicted number of circumcisers required depends on the assumed period during which full MAMC scale-up is achieved. Our base case assumption that a period of 5 years will be sufficient to circumcise most of the uncircumcised males might be seen as optimistic. The sensitivity analysis showed that the required number of circumcisers would be less with a longer start-up phase; however, the associated costs to scale up MAMC were relatively insensitive to this duration. Our assumption that a high fraction of uncircumcised men would accept circumcision may be considered an upper limit, reflecting the hypothesis that education campaigns promoting MAMC might even increase demand above levels reported in acceptability studies. Finally, our assumption about circumciser productivity is based on recent experience and may turn out to be conservative as efforts are made to design and evaluate more efficient surgical methods and ways of deploying staff.

Despite the consistent impacts found in the 3 RCTs, the exact impact that MAMC will have when scaled up in the field remains uncertain. For example, impact observations in these trials were all limited to the first 2 years after surgery. In addition, the indirect effect of circumcision on preventing mother-to-child transmission and associated reductions in medical costs were not taken into account in our model.

The mathematical model used to calculate epidemiological impact was a simple susceptible-infected compartmental model [9], which, for example, does not account for heterogeneity in HIV transmission by age. Our prediction of impact on HIV infections is similar to that of other HIV epidemic models [6], [9]. For example, our estimates of the number of MAMCs required to avert an HIV infection and program cost per HIV infection averted are consistent with a prior analysis using slightly different modelling assumptions [6]. Our impact estimate is higher than one study [17], with differences due mainly to epidemic severity and modelling time horizon. This consistency with past work, in combination with the robustness of our findings in multivariate sensitivity analysis, suggests that our results are likely to be reasonable predictions of epidemic impact and associated financial savings from HIV care averted.

Our analysis cannot be used to estimate precisely the difference in costs between a private and a public scenario, due to a lack of firm data on the cost of either scenario, especially with evidence of wide variation in private pricing. A pragmatic view suggests that each delivery approach has advantages and disadvantages. The main advantage of the private provider scenario is that it is immediately available: subsidies to private sector MAMC facilities by public or private donors will make this sector quickly operational. The disadvantage of the private sector approach is its likely higher long-term cost, the lack of private doctors in many rural areas threatening geographical equity in access and the insufficient number of doctors to fully cover the need for MAMC.

The main advantage of the public sector approach is its potentially lower cost in the longer term and potentially better geographic equity. The main disadvantage is that the health system may take time to make MC available on a large scale. The public system may require infrastructure development and training of health workers.

Summed over all the countries evaluated, the cost estimates for rolling out MAMC may appear to be high. We nevertheless think that this cost is affordable, for several reasons. First, the cost is high only for a few initial years: once most men have been circumcised, the cost will be reduced to the circumcision of men becoming adults (and eventually to newborns). Second, the cost is an investment which will prevent spending far greater resources in treating persons with HIV/AIDS in future years. Compared with our ART costing assumptions, the costs of ART may even increase if treatment initiation criteria are widened (to earlier stages of infection/disease) in coming years, due to longer therapy and an increasing need for more expensive second- and third-line regimens. Furthermore, spending for MC is modest compared with overall HIV control efforts: our prediction of annual funding required for MAMC roll-out for a 10-year period in the public provider scenario is only one-quarter of the current spending of the PEPFAR program: $433 million annually in 5 countries of Southern Africa [18]. The projected funding requirements for MAMC represent a significant and highly variable proportion of a country's total public and private health expenditures, estimated at 0.3%, 1.1%, 2.3% and 6.0% for South Africa, Tanzania, Zimbabwe and Botswana, respectively [19]. While this has important implications for planning and budgeting, it does not reflect on long-term affordability, since MAMC is cost saving.

We calculated that, over the first 5 years, about 1/4 full-time circumcisers would be required per 10 000 adults. Current general practitioners may be too few (especially those trained and willing to perform MC) and too busy to fulfil such a need [20], [21]. Furthermore, the training of general practitioners takes time and it is not reasonable to assume general practitioners will do just MC. Thus we believe that the training of nurses with an accreditation system could be a rapid way to increase the capacity of the private sector. The workforce shortage being the biggest barrier to roll out of MAMC [22], the involvement of nurses is likely to be a crucial step for an accelerated roll-out of MC. It will require some regulation adjustments because in many countries where MC is not common nurses are not allowed to perform MAMC, even in places where traditional circumcisers without medical knowledge and training are tolerated.

One of the main obstacles to the roll-out of MAMC is the relative technical difficulty of the surgery, which requires precise incisions, haemostasis and sutures. The roll-out of MAMC could be greatly facilitated and accelerated by the development of simplified, bloodless methods [23], which would lighten and shorten the training required for health workers and decrease costs. A full review of these bloodless methods and their acceptability in modern medical practice is therefore an urgent public health need [24]. In addition, we are exploring the applicability of “task specialization” team methods with substantially higher productivity per circumciser, similar to those pioneered for cataract removal surgery in Asia in the 1980s [25]–[27]. This approach can also make excellent use of low level health care workers for the less technical parts of the procedure (e.g., patient preparation and wound dressing).

A major consideration in scaling up MAMC is whether to concentrate on a horizontal or vertical approach. The horizontal approach is exemplified by the integration of MAMC into routine clinical practice. It is best represented in this analysis by the private sector scenario, since many general practitioners are likely to do MAMC as part of their varied clinical activities. The vertical approach makes MAMC a stand-alone activity. The public sector scenario may work with a vertical or horizontal emphasis, or a mix. The vertical approach offers potential to contribute uniquely to a rapid scale-up and the horizontal approach offers more structure for sustainability. We believe that a combination is preferred and that the optimal scale-up methods will depend on the health care system settings. We have not distinguished the cost of the public sector's horizontal versus vertical approaches, which will be the subject of future analyses.

The rapid implementation of MC will necessitate more than just funding. It will require broad involvement from many groups: national political leaders, activists, teachers, street leaders, churches and health workers. MAMC roll-out will also require strong and steady political support. The political involvement of South Africa will be key, as South Africa represents a high fraction of the population that could benefit the most and has a leading political role in the African region. Our hope is that the research done in the past 20 years regarding the potential of MC to reduce the spread of HIV will be recognized not merely as scientific progress, but as the foundation for an effective transition from knowledge to high-impact practice.

## Supporting Information

Supporting Information S1 Technical description of the modelling. Cost model of the roll-out of adult male circumcision in Africa integrated with a demographic and HIV epidemic model.
(0.15 MB DOC)

Figure S1 Compartment model of the modelled population
(0.06 MB TIF)
