# Supplementary material for: Estimating the Resources Needed and Savings Anticipated from Roll-Out of Adult Male Circumcision in Sub-Saharan Africa
Source: PLoS One. 2008 Aug 6;3(8):e2679. doi: 10.1371/journal.pone.0002679 (PMC2475667; doi:10.1371/journal.pone.0002679)
Supplement: Supporting Information S1 — Technical description of the modelling. Cost model of the roll-out of adult male circumcision in Africa integrated with a demographic and HIV epidemic model. (0.15 MB DOC) [file pone.0002679.s001.doc]

This document is a technical description of a mathematical model used to estimate the cost of the roll-out of male circumcision (MC) in those African countries where most of the males are currently uncircumcised. This model is based on an earlier epidemiological model [1] but now considers also the delivery of the intervention, cost parameters and details on antiretroviral therapy (ART).

**1 Introduction**

In a dynamic population composed of children and adults (see Figure S1), we consider the sub-population (P) of adults who are either HIV-negative or who have not yet developed AIDS (i.e. are still in the incubation period). People develop AIDS at the end of the incubation period: on reaching this endpoint, HIV-positive persons either die of AIDS or they receive ART. We assume that people receiving ART no longer transmit HIV and that they eventually die of AIDS or of other causes.

We divide the adult population (P) into 3 groups: Group 1 is composed of uncircumcised adult males: Group 2 of circumcised adult males: Group 3 of adult females. We assume that the sex ratio is 1. The adult population is characterised by its initial size (N_o_), mortality rate (K) due to causes other than AIDS, and U, the individuals reaching adulthood per unit time, expressed as a fraction of the adult population. A constant proportion (P_c_) of males is circumcised by traditional circumcisers before becoming adults.

The mortality K was calculated from the life expectancy when reaching adulthood (L_a_) in the absence of AIDS as $\text{K}=\frac{1}{\text{L}_{\text{a}}}$. If the birth rate (N_at_) and the fraction of newborns reaching the adult age (F_a_) are independent of time, the proportion of the total population becoming adult per unit of time is U = N_at_ F_a_/ R_a_ where R_a_ is the proportion of adults in the population.

We consider that this population is living in a particular geographical setting, which can be a district, a province, a country or a region. In this setting, funding (F), discounted at rate R, is used over period (D) to cover the direct costs of an intervention based on medical adult male circumcision (MAMC). These costs are composed of *initial* costs and *annual* costs. We model the intervention using *private* and *public* costing scenarios.

In the private cost model there is no initial cost. The annual costs (C_s_) are for the promotion of the intervention and the cost (C_mc_) of each MAMC. The latter cost, on average, covers the salary of circumcisers, patient counselling, surgical material, patient follow-up, running the circumcision unit and treating adverse events.

In the public cost model, the initial costs are the cost (C_u_) of establishing the infrastructure, medical equipment, the certification of the (N_u_) circumcision units where a number (D_c_) of circumcisers work (per unit), and the cost (C_c_) of the initial training of each circumciser. The annual costs comprise the continued promotion (C_s_) of the MAMC intervention in the modelled geographic setting, the annual salary (S_c_) of the circumcisers, and the cost (C_mc_) of each MAMC. This last cost, on average, covers patient counselling, surgical material, follow-up of patients, the running of the circumcision unit, and treatment of adverse events.

The analysis adopts the perspective of government health care. In the private cost model, the cost of each MAMC is the cost that the government health care will reimburse to private circumcisers. Costs are for the initial year, and future costs and benefits are discounted to 2007 at an annual discount rate (R). A_i_, the discount factor for year i, is defined as:

$$\text{A}_{\text{i}}=\frac{1}{\text{(1}+\text{R)}^{\text{i}-1}}$$

In the modelled population, there is no MAMC at the start, and during each year after the start of the intervention, a constant annual number (x) of MAMCs are performed. We assume that the men who get MAMC are not different with respect to infection status, age, or risk behaviour from the men who do not get MAMC. Once the cumulative number of MAMCs performed reaches a certain threshold, the prevalence of MC saturates and stabilizes. This defines the end of the so-called ***initial period of the intervention***. We assume that a proportion (P_no_) of males will not agree to be circumcised. After the initial period, the number of circumcisions (x_sat_) to be performed each year is the number of male adolescents entering adulthood, willing to be circumcised, and not already circumcised at birth or by traditional circumcisers.

**2 Demographics, HIV and circumcision models**

We have three groups of people: uncircumcised men (i=1), circumcised men (i=2) and

women (i=3). The size of each group is ni, the number that are HIV-positive is ni^+^, the HIV incidence is zi, and ai reach the end of the incubation period per unit time. The number of men who refuse circumcision is ni,n0.

We have the following set of equations **(set 1)**:

$$\begin{matrix} \frac{\text{dn}_{1}}{\text{dt}}=\text{U}\frac{\text{(n}_{1}+\text{n}_{2}+\text{n}_{3}\text{)}}{2}\text{(1}-\text{P}_{\text{c}}\text{)}-\text{Kn}_{1}-\text{a}_{1}-\text{x} \\ \frac{\text{dn}_{\text{1,no}}}{\text{dt}}=\text{U}\frac{\text{(n}_{1}+\text{n}_{2}+\text{n}_{3}\text{)}}{2}\text{P}_{\text{no}}-\text{Kn}_{\text{1,no}}-\text{a}_{\text{1,no}} \\ \frac{\text{dn}_{2}}{\text{dt}}=\text{U}\frac{\text{(n}_{1}+\text{n}_{2}+\text{n}_{3}\text{)}}{2}\text{P}_{\text{c}}-\text{Kn}_{2}-\text{a}_{2}+\text{x} \\ \frac{\text{dn}_{3}}{\text{dt}}=\text{U}\frac{\text{(n}_{1}+\text{n}_{2}+\text{n}_{3}\text{)}}{2}-\text{Kn}_{3}-\text{a}_{3} \\ \frac{\text{dn}_{1}^{+}}{\text{dt}}=\text{z}_{1}-(\text{n}_{1}^{+}-\text{n}_{\text{1,no}}^{+})\frac{\text{x}}{\text{n}_{1}-\text{n}_{\text{1,no}}}\text{-Kn}_{1}^{+}\text{-a}_{1} \\ \frac{\text{dn}_{\text{1,no}}^{+}}{\text{dt}}=\text{z}_{\text{1,no}}\text{-Kn}_{\text{1,no}}^{+}\text{-a}_{\text{1,no}} \\ \frac{\text{dn}_{2}^{+}}{\text{dt}}=\text{z}_{2}+(\text{n}_{1}^{+}-\text{n}_{\text{1,no}}^{+})\frac{\text{x}}{\text{n}_{1}-\text{n}_{\text{1,no}}}\text{-Kn}_{2}^{+}\text{-a}_{2} \\ \frac{\text{dn}_{3}^{+}}{\text{dt}}=\text{z}_{3}\text{-Kn}_{3}^{+}\text{-a}_{3} \end{matrix}$$

with the following formulae

$$\begin{matrix} \text{z}_{1}=\text{K}_{1}\text{(e}^{\beta\frac{\text{n}_{3}^{+}}{\text{n}_{3}}}\text{-1)(n}_{1}-\text{n}_{1}^{+}\text{)e}^{\text{-}\alpha\frac{\text{n}_{1}^{+}}{\text{n}_{1}}} \\ \text{z}_{\text{1,no}}=\text{K}_{1}\text{(e}^{\beta\frac{\text{n}_{3}^{+}}{\text{n}_{3}}}\text{-1)(n}_{\text{1,no}}-\text{n}_{\text{1,no}}^{+}\text{)e}^{\text{-}\alpha\frac{\text{n}_{\text{1,no}}^{+}}{\text{n}_{\text{1,no}}}} \\ \text{z}_{2}=\text{K}_{2}\text{(e}^{\beta\frac{\text{n}_{3}^{+}}{\text{n}_{3}}}\text{-1)(n}_{2}-\text{n}_{2}^{+}\text{)e}^{\text{-}\alpha\frac{\text{n}_{2}^{+}}{\text{n}_{2}}} \\ \text{z}_{3}=\text{K}_{3}\text{(e}^{\beta\frac{\text{n}_{1}^{+}}{\text{n}_{1}}}\text{-1)(n}_{3}-\text{n}_{3}^{+}\text{)}\frac{\text{n}_{1}}{\text{n}_{1}+\text{n}_{2}}\text{e}^{\text{-}\alpha\frac{\text{n}_{3}^{+}}{\text{n}_{3}}}+ \\ \text{K}_{4}\text{(e}^{\beta\frac{\text{n}_{2}^{+}}{\text{n}_{2}}}\text{-1)(n}_{3}-\text{n}_{3}^{+}\text{)}\frac{\text{n}_{2}}{\text{n}_{1}+\text{n}_{2}}\text{e}^{\text{-}\alpha\frac{\text{n}_{3}^{+}}{\text{n}_{3}}} \end{matrix}$$

We assumed that the incidence z in each group is proportional to: a) the number of HIV-negative people, b) a function of the proportion of HIV-positive people of the opposite gender characterised by the term containing β and c) a coefficient K which is a transmission coefficient. To allow for heterogeneity in sexual behaviour and to fit the observed asymptotic prevalence of infection, the transmission parameter takes the value K1, K2, K3 or K4 at the start of the epidemic and declines exponentially at rate α times the prevalence of infection.

We define

$$\begin{matrix} \text{K}_{2}=\text{QK}_{1} \\ \text{K}_{3}=\text{XK}_{1} \\ \text{K}_{4}=\text{Q'XK}_{1} \end{matrix}$$

In these last formulae, Q (≤1) is the effect of the circumcision on the rate of female-to-male transmission, X the ratio of transmission rates among uncircumcised-males-to-females to females-to-uncircumcised-males and Q' the effect of the circumcision on the rate of male-to-female transmission.

In Group 1 the number of HIV infections between the period of time between v and v+dv is z_1,v_ dv. From these infections, the number of people who should reach the end of the incubation period in the period between t and t+dt is n(v)= z_1,v_ dv W_t-v_ dt in the absence of cause of mortality other than AIDS and in the absence of circumcision. In this last expression, W is the probability density function of a Weibull distribution with shape parameter 2.25 and median 9.8 years. [1]

The group n(v) is composed of $\text{n}_{\text{a}}(\text{v})=\frac{\text{n}_{\text{1,no}}(\text{v})}{\text{n}_{1}(\text{v})}\text{n(v)}$ people who refuse to be circumcised and of $\text{n}_{\text{b}}\text{(v)}=\text{(1}-\frac{\text{n}_{\text{1,no}}(v)}{\text{n}_{1}(v)}\text{)n(v)}$who agree to be circumcised.

For simplicity we omit below the explicit dependence of the variables on v and t.

In the period between v and t, n_a_ obeys the following differential equation:

$$\frac{\text{d}n_{a}}{dt}=-\text{Kn}_{\text{a}}$$

which gives

$\text{n}_{a}=\frac{\text{n}_{\text{1,no}}}{\text{n}_{1}}\text{ne}^{-\text{K(t-v)}}$.

In the same period, n_b_ obeys the differential equation

$$\frac{\text{dn}_{\text{b}}}{dt}=-\text{Kn}_{\text{b}}-\frac{\text{x}}{\text{n}_{1}-\text{n}_{\text{1,no}}}\text{n}_{\text{b}}$$

which gives

$$\text{n}_{\text{b}}=\text{(1}-\frac{\text{n}_{\text{1,no}}}{\text{n}_{1}}\text{)ne}^{-\text{K(t-v)}}\text{e}^{-\int_{\text{v}}^{\text{t}} \frac{\text{x}}{\text{n}_{1}-\text{n}_{\text{1,no}}}\text{dt'}}$$

It follows that

$$\text{n}=\frac{\text{n}_{\text{1,no}}}{\text{n}_{1}}\text{z}_{\text{1,v}}\text{dvW}_{\text{t-v}}\text{e}^{-\text{K(t-v)}}\text{dt}+\text{(1}-\frac{\text{n}_{\text{1,no}}}{\text{n}_{1}}\text{)z}_{\text{1,v}}\text{dvW}_{\text{t-v}}\text{e}^{-\text{K(t-v)}}\text{e}^{-\int_{\text{v}}^{\text{t}} \frac{\text{x}}{\text{n}_{1}-\text{n}_{\text{1,no}}}\text{dt'}}\text{dt}$$

When v varies between $-\infty$ and t, this number becomes

$$n=\left[\int_{\text{v}=\text{-}\infty}^{\text{t}} \frac{\text{n}_{\text{1,no}}}{\text{n}_{1}}\text{z}_{\text{1,v}}\text{W}_{\text{t-v}}\text{ e}^{-\text{K(t-v)}}\text{dv}\right]\text{dt}+\left[\int_{\text{v}=\text{-}\infty}^{\text{t}} \text{(1}-\frac{\text{n}_{\text{1,no}}}{\text{n}_{1}}\text{)} \text{z}_{\text{1,v}}\text{W}_{\text{t-v}}\text{ e}^{-\text{K(t-v)}}\text{e}^{-\int_{\text{v}}^{\text{t}} \frac{\text{x}}{\text{n}_{1}-\text{n}_{\text{1,no}}}\text{dt'}}\text{dv}\right]\text{dt}$$

Defining new variables $t-v=\tau$ and $t-t'=u$, we have:

$$\begin{matrix} n=\left[\int_{\text{τ}=0}^{\infty} \left(\frac{\text{n}_{\text{1,no}}}{\text{n}_{1}}\right)_{\text{t-τ}}\text{z}_{\text{1,t}-\text{τ}}\text{e}^{-\text{Kτ}}\text{W}_{\text{τ}}\text{dτ}\right]\text{dt} \\ +\left[\int_{\text{τ}=0}^{\infty} \left(1-\frac{\text{n}_{\text{1,no}}}{\text{n}_{1}}\right)_{\text{t-τ}}\text{z}_{\text{1,t}-\text{τ}}\text{e}^{-\text{Kτ}}\text{e}^{-\int_{0}^{\text{τ}} \left(\frac{\text{x}}{\text{n}_{1}-\text{n}_{\text{1,no}}}\right)_{\text{t-u}}\text{du}}\text{W}_{\text{τ}}\text{dτ}\right]\text{dt} \end{matrix}$$

It follows that a_1_, a_2_ and a_3_ are given by the following equations **(set 2)**:

$$\begin{matrix} \text{a}_{1}=\int_{0}^{\infty} \left(\frac{\text{n}_{\text{1,no}}}{\text{n}_{1}}\right)_{\text{t-τ}}\text{z}_{\text{1,t}-\text{τ}}\text{e}^{-\text{Kτ}}\text{W}_{\text{τ}}\text{dτ}+\int_{0}^{\infty} \left(1-\frac{\text{n}_{\text{1,no}}}{\text{n}_{1}}\right)_{\text{t-τ}}\text{z}_{\text{1,t}-\text{τ}}\text{e}^{-\text{Kτ}}\text{e}^{-\int_{0}^{\text{τ}} \left(\frac{\text{x}}{\text{n}_{1}-\text{n}_{\text{1,no}}}\right)_{\text{t-u}}\text{du}}\text{W}_{\text{τ}}\text{dτ} \\ \text{a}_{\text{1,no}}=\int_{0}^{\infty} \left(\frac{\text{n}_{\text{1,no}}}{\text{n}_{1}}\right)_{\text{t-τ}}\text{z}_{\text{1,t}-\text{τ}}\text{e}^{-\text{Kτ}}\text{W}_{\text{τ}}\text{dτ} \\ \text{a}_{2}=\int_{0}^{\infty} \text{z}_{\text{2,t}-\text{τ}}\text{e}^{-\text{Kτ}}\text{W}_{\text{τ}}\text{dτ}+\int_{0}^{\infty} \left(1-\frac{\text{n}_{\text{1,no}}}{\text{n}_{1}}\right)_{\text{t-τ}}\text{z}_{\text{1,t}-\text{τ}}\text{e}^{-\text{Kτ}}\left[\text{1-e}^{-\int_{0}^{\text{τ}} \left(\frac{\text{x}}{\text{n}_{1}-\text{n}_{\text{1,no}}}\right)_{\text{t-u}}\text{du}}\right]\text{W}_{\text{τ}}\text{dτ} \\ \text{a}_{3}=\int_{0}^{\infty} \text{z}_{\text{3,t}-\text{τ}}\text{e}^{-\text{Kτ}}\text{W}_{\text{τ}}\text{dτ} \end{matrix}$$

In each group, the HIV prevalences p_1_, p_2_ and p_3_, and the HIV incidence ratios f_1_, f_2_ and f_3_ are given by

$$\begin{matrix} \text{p}_{1}=\frac{\text{n}_{1}^{+}}{\text{n}_{1}} \\ \text{p}_{2}=\frac{\text{n}_{2}^{+}}{\text{n}_{2}} \\ \text{p}_{3}=\frac{\text{n}_{3}^{+}}{\text{n}_{3}} \\ \text{f}_{1}=\frac{\text{z}_{1}}{\text{n}_{1}-\text{n}_{1}^{+}} \\ \text{f}_{2}=\frac{\text{z}_{2}}{\text{n}_{2}-\text{n}_{21}^{+}} \\ \text{f}_{3}=\frac{\text{z}_{3}}{\text{n}_{3}-\text{n}_{3}^{+}} \end{matrix}$$

Averaged over the three groups, the HIV prevalence (p) and the incidence ratio (f) are given by:

$$\begin{matrix} \text{p}=\frac{\text{n}_{1}^{+}+\text{n}_{2}^{+}+\text{n}_{3}^{+}}{\text{n}_{1}+\text{n}_{2}+\text{n}_{3}} \\ \text{f}=\frac{\text{z}_{1}+\text{z}_{2}+\text{z}_{3}}{\text{n}_{1}\text{(1}-\text{p}_{1}\text{)}+\text{n}_{2}\text{(1}-\text{p}_{2}\text{)}+\text{n}_{3}\text{(1}-\text{p}_{3}\text{)}} \end{matrix}$$

**4 Saturation level**

If x is sufficiently large, n1 decreases with time and the proportion of circumcised males $\text{y}=\frac{\text{n2}}{\text{n1}+\text{n2}}$ saturates.

We call x_sat_ the number of circumcisions required per unit of time to maintain this level. In this saturation state, we assumed that circumcisions are performed soon after males become adults, before any possible HIV infection. Under this assumption the only uncircumcised men are those who refused to be circumcised; thus $\text{n}_{1}\text{-n}_{\text{1,no}}=0$

x_sat_ is given by the equation $\frac{\text{d(n}_{1}\text{-n}_{\text{1,no}}\text{)}}{dt}=0$. It follows:

$$\text{x}_{\text{sat}}=\text{U}\frac{\text{(n}_{1}+\text{n}_{2}+\text{n}_{3}\text{)}}{2}\text{(1}-\text{P}_{\text{c}}-\text{P}_{\text{no}}\text{)}-\text{K(n}_{1}-\text{n}_{\text{1,no}})-(\text{a}_{1}-\text{a}_{\text{1,no}})$$

The equations giving $\frac{\text{dn}_{1}}{\text{dt}}\text{,} \frac{\text{dn}_{2}}{\text{dt}}\text{,} \frac{\text{dn}_{1}^{+}}{\text{dt}} \text{and} \frac{\text{dn}_{2}^{+}}{\text{dt}}$become:

$$\begin{matrix} \frac{\text{dn}_{1}}{\text{dt}}=\frac{\text{dn}_{\text{1,no}}}{\text{dt}} \\ \frac{\text{dn}_{2}}{\text{dt}}=\text{U}\frac{\text{(n}_{1}+\text{n}_{2}+\text{n}_{3}\text{)}}{2}\text{P}_{\text{c}}-\text{Kn}_{2}-\text{a}_{2}+\text{x}_{\text{sat}} \\ \frac{\text{dn}_{1}^{+}}{\text{dt}}=\text{z}_{1}\text{-Kn}_{1}^{+}\text{-a}_{1} \\ \frac{\text{dn}_{2}^{+}}{\text{dt}}=\text{z}_{2}\text{-Kn}_{2}^{+}\text{-a}_{2} \end{matrix}$$

The other equations remain unchanged.

**5 State at origin**

We estimated the initial HIV prevalence among circumcised and uncircumcised men, and among women, by running the model for a given number of years (typically 100 years) from a low HIV prevalence (1 %). We also used this initial run to determine the HIV incidence in each group in the years prior to the year t=0. These values are needed to calculate the number of AIDS cases for t>0.

**6 Generation of results**

The sets 1 and 2 were discretised with a time step of T and implemented using Excel. We set α = 9, giving a reasonable initial doubling time of 2.9 years for the epidemic.

The value of K1 was adjusted to obtain the desired value of HIV prevalence when a steady state was reached before the MC intervention.

The MC effect was adjusted to obtain a given reduction of HIV prevalence following 100% of MC coverage after reaching a new steady state.

**7 Funding spent and consequences**

In the private cost model, the discounted annual funding spent (F_s_) is given as a function of time and of the number of required circumcisers (n_c_) by the following three expressions:

At year 1,

$$\text{F}_{\text{s}}\text{(1)}=\text{C}_{\text{s}}+\text{xC}_{\text{mc}}$$

At any year (y>1) before reaching the saturation level:

$$\text{F}_{\text{s}}\text{(y)}=\text{(C}_{\text{s}}+\text{xC}_{\text{mc}}\text{)A}_{\text{y}}$$

After reaching the saturation level:

$$\text{F}_{\text{s}}\text{(y}>\text{y}_{\text{sat}}\text{)}=\text{(C}_{\text{s}}+\text{x}_{\text{sat}}\text{C}_{\text{mc}}\text{)A}_{\text{y}}$$

In the public cost model, the discounted annual funding spent is given as a function of time and of the number of required circumcisers (n_c_) by the following three expressions:

At year 1,

$$\text{F}_{\text{s}}\text{(1)}=\text{C}_{\text{s}}+\text{N}_{\text{u}}\text{C}_{\text{u}}+\text{xC}_{\text{mc}}+\text{n}_{\text{c}}\text{(C}_{\text{c}}+\text{S}_{\text{c}}\text{)}$$

At any year (y>1) before reaching the saturation level:

$$\text{F}_{\text{s}}\text{(y)}=\text{(C}_{\text{s}}+\text{xC}_{\text{mc}}+\text{n}_{\text{c}}\text{S}_{\text{c}}\text{)A}_{\text{y}}$$

After reaching the saturation level:

$$\text{F}_{\text{s}}\text{(y}>\text{y}_{\text{sat}}\text{)}=\text{(C}_{\text{s}}+\text{x}_{\text{sat}}\text{C}_{\text{mc}}+\text{n}_{\text{c}}\text{S}_{\text{c}}\text{)A}_{\text{y}}$$

We considered the cost (V_1_) of treating HIV-positive people before they become eligible for ART, the average cost (V_2_) of one year of antiretroviral treatment (ART) per person and the cost (V_3_) of treating HIV-positive people after becoming eligible for ART with non-ART therapy. We calculated the cost of ARTs (see below) as a function of time and we defined the **cost equilibrium period** as the period needed for the cumulative funding spent for the intervention to be equal to the cumulative cost of averted ARTs during the same period of time.

The number (x) of circumcisions per year before reaching the saturation level is assumed to be a constraint of the implementation of the intervention. The period of time taken to reach the saturation level decreases as x increases. Spending is given by the following equation

$\text{F}=\sum_{1}^{\text{D}} \text{Fs(t)}$ with$\text{Nc}=\frac{\text{x}}{ \text{N}}$, N being the number of circumcisions that a circumciser can do per unit of time. This number becomes $\text{Nc}=\frac{\text{x}_{\text{sat}}}{ \text{N}}$ after reaching the saturation level. The number of required circumcision units is given by$\frac{\text{N}_{\text{c}}}{ \text{Dc}}$.

The number of HIV cases averted each year as a function of time is given by:

$$\text{n}_{\text{a}}=\text{(z1}+\text{z2}+\text{z3)}_{\text{x}=0}-\text{(z1}+\text{z2}+\text{z3)}$$

Where the (z_i_)_x=0_ represent the predictions of a model with no MAMC.

Subsequently the cost per HIV infection averted is given by:

$$\frac{\sum_{1}^{\text{D}} \text{F}_{\text{s}}\text{(t)}}{\sum_{1}^{\text{D}} \text{n}_{\text{a}}}$$

The number of averted AIDS cases d_i_ at year i is given by:

$$\text{d}_{\text{i}}=\sum_{\tau=1}^{\text{S}} \text{ε}_{\text{τ}}\text{n}_{\text{a,i-}\tau}\text{W}_{\tau}\text{e}^{\text{-K}\tau}$$

The number of deaths averted each year was calculated as (1-Z)d_i_ with Z being the proportion of HIV-positive persons eligible for ART who effectively receive ART.

The cost of averted treatment for an HIV-positive person before becoming eligible for ART and of averted ART and non-ART therapy at each year i was calculated as

$$\text{A}_{\text{i}}\text{(d}_{\text{i}}\text{Z}_{1}\text{V}_{1}+\text{d}_{\text{i}}\text{Z}_{3}\text{V}_{3}+\text{Z}_{2}\text{V}_{2}\sum_{\text{k}=1}^{\text{H}} \text{d}_{\text{i}-\text{k}})$$

with H being the life expectancy of HIV-positive individuals when starting ART.

The saving of year i was then calculated as the difference between the funding spent and the cost of averted ARTs.

Reference

**1. Williams, B.G., J.O. Lloyd-Smith, E. Gouws, C. Hankins, W.M. Getz, J. Hargrove, I. de Zoysa, C. Dye, and B. Auvert*, The Potential Impact of Male Circumcision on HIV in Sub-Saharan Afric*a. PLoS Med, 2006. 3(7): p. e262.**
