# Supplementary figures and images for: Estimating the Resources Needed and Savings Anticipated from Roll-Out of Adult Male Circumcision in Sub-Saharan Africa
Source: PLoS One. 2008 Aug 6;3(8):e2679. doi: 10.1371/journal.pone.0002679 (PMC2475667; doi:10.1371/journal.pone.0002679)

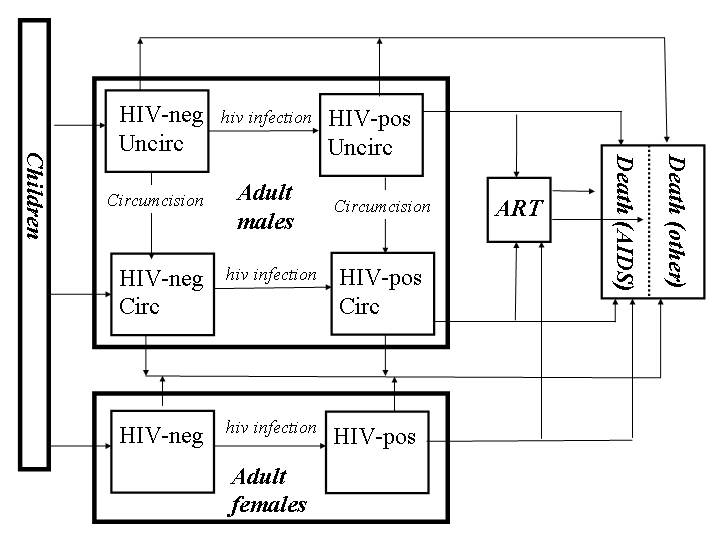

Supplement: Figure S1 — Compartment model of the modelled population (0.06 MB TIF) [file pone.0002679.s002.tif]
